# Distant Relatives of Severe Acute Respiratory Syndrome Coronavirus and Close Relatives of Human Coronavirus 229E in Bats, Ghana

**DOI:** 10.3201/eid1509.090224

**Published:** 2009-09

**Authors:** Susanne Pfefferle, Samuel Oppong, Jan Felix Drexler, Florian Gloza-Rausch, Anne Ipsen, Antje Seebens, Marcel A. Müller, Augustina Annan, Peter Vallo, Yaw Adu-Sarkodie, Thomas F. Kruppa, Christian Drosten

**Affiliations:** Bernhard Nocht Institute for Tropical Medicine, Hamburg, Germany (S. Pfefferle, J.F. Drexler, T.F. Kruppa); University of Bonn Medical Centre, Bonn, Germany (S. Pfefferle, J.F. Drexler, M.A. Müller, C. Drosten); Kwame Nkrumah University of Science and Technology, Kumasi, Ghana (S. Oppong, Y. Adu-Sarkodie); Noctalis, Centre for Bat Protection and Information, Bad Segeberg, Germany (F. Floza-Rausch, A. Ipsen, A. Seebens); Kumasi Centre for Collaborative Research in Tropical Medicine, Kumasi (A. Annan, T.F. Kruppa); Academy of Sciences of the Czech Republic, Brno, Czech Republic (P. Vallo)

**Keywords:** Bats, coronavirus, viruses, severe acute respiratory syndrome, human coronavirus, molecular clock, Ghana, research

## Abstract

We tested 12 bat species in Ghana for coronavirus (CoV) RNA. The virus prevalence in insectivorous bats (n = 123) was 9.76%. CoV was not detected in 212 fecal samples from *Eidolon helvum* fruit bats. Leaf-nosed bats pertaining to *Hipposideros ruber* by morphology had group 1 and group 2 CoVs. Virus concentrations were <45,000 copies/100 mg of bat feces. The diversified group 1 CoV shared a common ancestor with the human common cold virus hCoV-229E but not with hCoV-NL63, disputing hypotheses of common human descent. The most recent common ancestor of hCoV-229E and GhanaBt-CoVGrp1 existed in ≈1686–1800 ad. The GhanaBt-CoVGrp2 shared an old ancestor (≈2,400 years) with the severe acute respiratory syndrome–like group of CoV.

## Abstract 2

*Hipposideros* spp. bats harbor a coronavirus that shares common ancestry with human viruses.

Coronaviruses (CoVs) (order Nidovirales, family *Coronaviridae*, genus *Coronavirus*) are enveloped viruses with plus-stranded RNA genomes of 26–32 kb, the largest contiguous RNA genomes in nature (*1*). They are classified into 3 groups, which contain viruses pathogenic for mammals (groups 1 and 2) and poultry (group 3) (*1*). Human CoVs (hCoVs)-229E, -NL63, -OC43, and -HKU1 are endemic worldwide and cause mainly respiratory infections in children and adults. The severe acute respiratory syndrome (SARS) coronavirus (SARS-CoV) is a novel zoonotic coronavirus that caused an international epidemic in 2002–2003. Fortunately, efficient public health management interrupted this epidemic (*2*). Studies conducted in China in the aftermath of the SARS epidemic have identified CoVs in bats (Chiroptera) and implicated this speciose mammalian order as the most likely reservoir of all known coronaviruses (*3**–**7*). Among the most urgent concerns prompted by the SARS epidemic is the likelihood of similar future events. Thus, it seems highly relevant to study the ecology of bat CoVs in terms of diversity, host restriction, virus prevalence, risk of exposure, and the circumstances of past host transition events.

The genetic diversity of bat-borne CoVs is currently unclear. Preliminary data suggest that CoVs may be adapted in a stricter sense to a specific host species rather than to specific regions (*5**,**6**,**8**–**12*). A variety of pathogenic CoVs occur in other mammals or poultry. However, the genetic range within these animals is considerably less than that observed in even single bat species or subfamilies (*7**,**8*).

Estimates indicate that there are >100 bat species in sub-Sahran Africa. This finding is in contrast to ≈50 species in the entire Western Palaearctic region (Europe, Middle East, North Africa) (*13**,**14*). African bats have been shown to harbor pathogens that are occasionally transmitted to humans. This transmission may result in severe disease outbreaks, e.g., Ebola and Marburg viruses (*15*). Because bats are a part of the human diet in wide areas of Africa (*16*), it appears highly relevant to study CoVs in African bats.

We have demonstrated by serologic studies that African bats have antibodies against CoVs (*10*). Antibodies reactive with SARS-CoV antigen were detected in 47 (6.7%) of 705 bat serum specimens from 26 species (*10*). Recently, Tong et al. detected sequences of CoVs in bats from Kenya (*17*). We describe the results of studies on bats in Ghana obtained by using noninvasive sampling of frugivorous and insectivorous bats at 2 caves, a lake habitat of diverse insectivorous bats, and a large urban roosting site of frugivorous bats. Bayesian inference of diversification dates gave implications on the recency of the introduction of hCoV-229E into the human population, irrespective of its original source.

## Materials and Methods

### Capturing and Sampling

**Figure 1:**
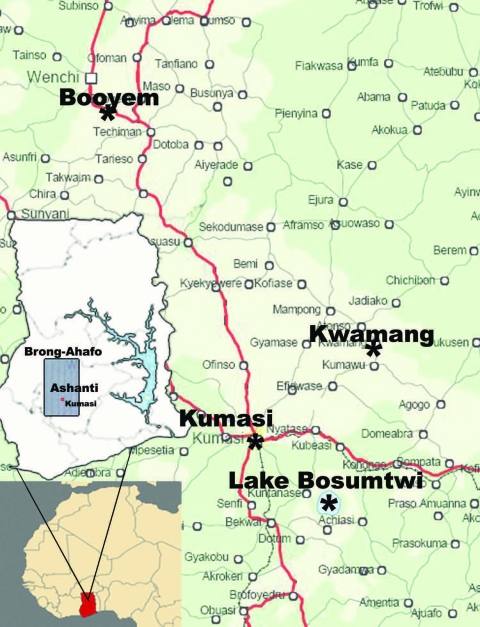
Location of Kwamang caves near the village of Kwamang, (6°58′N, 1°16′W), 50 km northeast of Kumasi, Ashanti region, Ghana. Booyem caves A (7°43′24.9′′N, 1°59′16.5′W) and B (7°43′25.7′′N, 1°59′33.5′′W) are located near remote small settlements in the vicinity of Booyem, Brong-Ahafo region. Lake Bosumtwi is located 30 km southeast of Kumasi (6°32′22.3′′N, 1°24′41.5′′W). The botanical gardens of Kwame Nkrumah National University of Science and Technology are located on campus in the city of Kumasi (6°41′6.4′′N, 1°33′42.8′′W). Kumasi Zoo is located in the center of the city (6°42′2.0′′N, 1°37′29.9′′W).

In the locations identified in Figure 1, mist netting and sampling were conducted as described (*11*). In Kumasi Zoo, fecal samples were collected with plastic foil under trees occupied by *Eidolon helvum* bats (estimated colony size 300,000). For all capturing and sampling, permission was obtained from the Wildlife Division of the Ministry of Lands, Forestry, and Mines in Ghana. Research samples were exported under a state agreement between the Republic of Ghana and the Federal Republic of Germany, represented by the City of Hamburg. Additional export permission was obtained from the Veterinary Services of the Ghana Ministry of Food and Agriculture.

### Processing and Analysis of Samples

Samples (1–4 fecal pellets or swabs suspended in RNA stabilization solution [RNAlater Tissue Collection; Applied Biosystems, Foster City, CA, USA]) were tested at the Kumasi Centre for Collaborative Research in Tropical Medicine as described (*11**,**18*). After initial sequencing, specific primers were designed for each group of CoV found. Nested reverse transcription–PCR (RT-PCR) primer sets used for sequencing of longer fragments of representative viruses are available upon request. The following sequences were derived from this study and were submitted to GenBank under the listed accession numbers: RNA-dependent RNA polymerase (RdRp) sequences: BtCoV/Hip/GhanaBoo/348/2008, FJ710043; BtCoV/Hip/GhanaBoo/344/2008, FJ710044; BtCoV/Hip/GhanaKwam/8/2008, FJ710045; BtCoV/Hip/GhanaKwam/19/2008, FJ710046; BtCoV/Hip/GhanaKwam/20/2008, FJ710047; BtCoV/Hip/GhanaKwam/13/2008, FJ710048; BtCoV/Hip/GhanaKwam/31/2008, FJ710049; BtCoV/Hip/GhanaKwam/27/2008, FJ710050; BtCoV/Hip/GhanaKwam/26/2008, FJ710051; BtCoV/Hip/GhanaKwam/24/2008, FJ710052; BtCoV/Hip/GhanaKwam/10/2008, FJ710053; BtCoV/Hip/GhanaKwam/22/2008, FJ710054/nucleocapsid sequences; BtCoV/Hip.sp/GhanaBoo/344/2008, FJ710055; BtCoV/Hip.sp/GhanaKwam/19/2008, FJ710056.

### Phylogenetic Analysis

Nucleic acid alignments were conducted based on amino acid code by using the ClustalW algorithm (www.ebi.ac.uk/clustalw) in the Molecular Evolutionary Genetics Analysis version 4.0 software package (www.megasoftware.net) (*19*). Two gap-free nucleotide alignments (817 bp and 1,221 bp) were generated. Tree topologies were determined on both datasets by using MrBayes version 3.1 (*20*). The analysis used a general time reversible (GTR) substitution model with 6 rate categories to approximate a gamma-shaped rate distribution across sites and an invariant site assumption (GTR + Γ6 + I). Markov chain Monte Carlo (MCMC) chains of 10^7^ iterations were sampled every 500 generations, resulting in 20,000 sampled trees. Two Metropolis-coupled chains (1 cold and 3 heated chains each) were run in parallel, compared, and pooled. Convergence of chains was confirmed by the potential scale reduction factor statistic in MrBayes (*21*) and by visual inspection of each cold chain using the TRACER program (*22*). Phylogenetic dating was conducted by using Bayesian evolutionary analysis sampling trees (BEAST) (*22*). Chain lengths in BEAST were at least 20,000,000 generations with sampling every 500 generations. Convergence of the model was checked visually and by the effective sample size statistic with TRACER.

## Results

### Virus Detection

During February 2008, bats were sampled in the described locations around Kumasi, Ghana. Initially, 7 fecal samples tested positive by pan-CoV PCR. Products (440 bp, *RdRp* gene) were sequenced and aligned with prototype CoV. Neighbor-joining phylogenies indicated 2 distinct groups of sequences that belonged to CoV group 1 (n = 4) and group 2 (n = 3), respectively. Specific primer pairs for the group 1 and group 2 sequences were designed and applied again to all samples. Five additional viruses were found, resulting in a total CoV prevalence of 9.76% in insect-eating bats (n = 123). No virus was found in any oral swab. All virus findings in fecal samples are listed by capture site in Table 1.

**Table 1:** Overview of bats studied, Ghana

Sampling site	Species	No. fecal samples	No. positive (group 1/group 2)
Zoo Kumasi (6°42′2.0′′N, 1°37′29.9′′W)	*Eidolon helvum*	212*	0
KNUST Botanical Garden Kumasi (6°41′6.4′′N, 1°33′42.8′′W)†	*Pipistrellus nanulus*	1	0
	*Glauconycteris beatrix*	1	0
Lake Bosumtwi (6°32′22.3′′N, 1°24′41.5′′W)	*Chaerephon spp.*	6	0
	*Nycteris hispida*	1	0
	*P. nanus*	5	0
	*P. deserti*	1	0
Cave Kwamang (6°58′N, 1°16′W)	*Hipposideros caffer ruber*	40	10 (4/6)
	*H. abae*	13	0
Cave Booyem A (7°43′24.9′′N, 1°59′16.5′′W)	*H. cf. ruber*	8	0
	*Coleura afra*	12	0
Cave Booyem B (7°43′25.7′′N, 1°59′33.5′′W)	*H. cf. ruber*‡	11	2 (1/1)
	*H. abae*	3	0
	*Coleura afra*	21	0
Total		335	12

*These samples were collected without individual association to bats. Due to a low sampling frequency, it can be assumed that each sample was from an individual bat. †Kwame Nkrumah University of Science and Technology. ‡Two morphotypes were observed.

**Figure 2:**
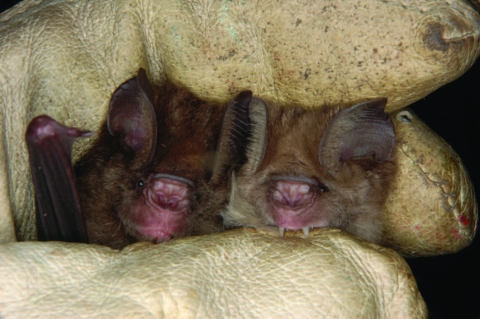
Two morphotypes of *Hipposideros* *caffer* *ruber* bats held by one of the authors (F.G.-R.), who was wearing a leather glove. Photograph courtesy of Antje Seebens.

Notably, all CoV findings were in insect-eating leaf-nosed bats of the genus *Hipposideros*. Within the genus, the species *H. abae* could be discriminated unambiguously by morphology (Table 1). The remaining *Hipposideros* species were assigned to the complex of forms related to currently recognized species *H. caffer* and *H. ruber.* Because 2 morphotypes were present (Figure 2), the mitochondrial cytochrome *b* gene was sequenced as described (*23*). Both morphotypes belonged to phylogenetic lineages distinct from *H. caffer* and possibly represented 2 distinct species (P. Vallo, personal ongoing investigation). Both are collectively referred to as *H. caffer* (*cf*.) *ruber* in this study. A fraction of 15.4% of *H. cf. ruber* specimens yielded CoV, without a difference between sexes (14%/19%, n = 57/21 [M/F], respectively). Only adult males and nonlactating adult females, but no lactating females, juveniles, and subadults of *H. cf. ruber* were encountered.

### Virus Concentrations

To estimate the quantity of CoV genomes in bat feces, we did end-point dilution experiments with the nested pan-CoV RT-PCR (*18*). The previously determined sensitivity limit of the PCR assay was 5–45 copies/PCR (*18*). In the assay, the equivalent of 1 mg feces was tested per PCR tube (100 mg feces collected, 1:10 dilution extracted, 1:10 dilution tested). The highest dilution factor that still yielded an amplification signal in any of the samples was 1:10, which suggested a maximal concentration of 50 to 450 CoV RNA copies/mg of feces.

### Virus Classification

#### Group 1 CoV

**Figure 3:**
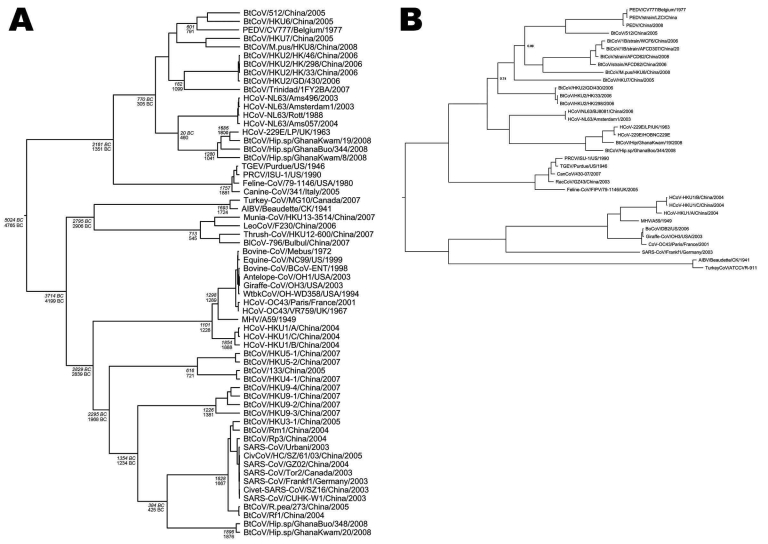
A) Phylogeny of coronaviruses (CoVs) in the RNA-dependent RNA polymerase gene (RdRp, 817-bp fragment) with root point dates derived from Bayesian inference under a relaxed lognormal molecular clock assumption with a codon-based substitution model (SRD06) and an assumption of expansion growth of the virus population. Estimated dates of diversification of CoV lineages at root points are shown in italics for the expansion growth population model and in regular type for the exponential growth model. Dates bc are identified with a suffix; dates ad are not. B) Bayesian phylogeny from the CoV group 1 root, using the nucleocapsid (N) gene. Highest posterior densities for all root points were >0.99, except where indicated.

In *H. cf. ruber* bats in the Kwamang and Booyem caves, a diverse group 1 CoV was found. Further analysis was complicated by the low RNA content in samples. Based on alignments of prototype group 1 viruses, 5 different nested RT-PCRs were designed and the *RdRp* fragment could finally be extended by 441 bp to the 5′ end, providing an 817-nt fragment for phylogenetic analysis. All methods of phylogenetic inference placed this virus next to a common ancestor with human coronavirus 229E, which circulates worldwide in humans (Figure 3). Bootstrap support of the hCoV-229E/GhanaBt-CoVGrpI root point in neighbor-joining analysis was 100%. The corresponding Bayesian posterior probability was 1.0. The most closely related member of the GhanaBt-CoVGrp1 clade shared 91.90% nucleotide identity with hCoV-229E in the analyzed fragment. The most distant member was 86.50% identical. The next phylogenetic neighbor, the human CoV hCoV-NL63, was only 74.70%–78.60% identical in the analyzed fragment.

#### Group 2 CoV

With the pan-CoV screening assay, a group 2 CoV was initially found in the Kwamang cave. Sequences from 3 bats were identical. The secondary group-specific PCR identified 4 additional samples of this virus, 1 of them from Booyem Cave B and the remaining from Kwamang. Nucleotide identity among these sequences was 97.2%–100%. Phylogenetic analysis with different methods of inference (neighbor-joining nucleotide-based, neighbor-joining amino acid–based, Bayesian) yielded variable tree topologies suggesting basal associations with either the 2a, 2d, or 2b subgroups (data not shown) (*24*). Based on alignments of prototype group II viruses, 8 additional nested RT-PCR primer sets were designed and 2 of the samples could be amplified. Sequences could be extended 520 bp upstream and 383 bp downstream of the initial fragments, yielding 1,221-bp fragments for phylogenetic analysis. Bayesian phylogenetic inference with different substitution models and parallel analysis using Metropolis coupling now placed the virus reliably next to a common ancestor with the 2b group of CoV (SARS-like viruses, Figure 3). The Bayesian posterior probability of the CoV 2b/GhanaBt-CoVGrp2 clade being monoyphletic was 1.0. A maximum of 72.2% nucleotide identity was shared with SARS CoV.

### Molecular Dating

Reliable isolation dates were researched in the literature for each employed virus. Because a reliable molecular clock dating existed for the most recent common ancestor (MRCA) of the hCoV-OC43/bovine CoV pair (*25*), this date was set as a normal-distributed probabilistic prior within the published ranges (*25*) for calibration of all analyses. A first analysis was conducted on the 1,225-bp dataset that did not include the novel GhanaBt-CoVGrp1. All virus sequences were assumed to be contemporary. Phylogeny was inferred using a GTR + Γ4 + I model. The resulting MRCA date of the CoV2b (SARS-like)/GhanaBt-CoVGrp2 clade was 260 ad and that of the hCoV-NL63/-229E pair was 981 ad (see Table 2 for details). To include the novel GhanaBat-CoVGrp1, we repeated the same analysis by using the 817-bp dataset. The resulting MRCA date of the hCoV-NL63/229E pair was 816 ad in this analysis, which was in good concordance with results from the 1,221-bp dataset (Table 2) and also with previously published data (*26*). The diversification estimate for the novel group 1 bat-CoV and hCoV-229E then was 1803 ad.

**Table 2:** Results of molecular clock analyses, study of coronaviruses in bats, Ghana*

Alignment, bp	Mean substitution rate (substitutions/ site/year)	Population model, substitution model	Root point (range)†	MRCA (95% CI, HPD)‡			
				SARS-like§	SARS-like/ GhanaBt-CoVGrp2	hCoV-229E/ hCoV-NL63	HCoV-229E/ GhanaBt-CoVGrp1
1,221	2.1 × 10^–4^ (1.2–3.1 × 10^–4^)	Constant size, nucleotide (GTR + G + I)	2243 (4521–290)	1905 ad (1867–1941)	260 (792 bc–1178)	981 (161 bc–1324)	–
817	2.1 × 10^–4^ (1.5–2.7 × 10^–4^)	Constant size, nucleotide (GTR + G + I)	2053 (3433–731 )	1852 ad (1612–1852)	348 (119 bc–820)	816 (320–1290)	1803 (1694–1906)
817	1.6 ×10^–4^ (0.8–2.5 × 10^–4^)	Constant size, codon-based (SRD06)	4500 (7305–1918)	1674 (1516–1804)	768 bc (2037 bc–360)	168 (1111 bc–721)	1659 (1503–1804)
817	1.5 × 10^–4^ (0.9–2.2 × 10^–4^)	Expansion growth, codon-based (SRD06)	5024 (9261–1360)	1628 (1379–1836)	384 bc (2060 bc–1074)	20 (1347 bc–1174)	1686 (1460–1871)
817	1.8 × 10^–4^ (0.9–2.8 × 10^–4^)	Exponential growth, codon-based (SRD06)	4765 (7999–1707)	1667 (1436–1853)	425 (1544 bc–1193)	460 (956 bc–1271)	1800 (1501–1883)

*MRCA, most recent common ancestor; CI, confidence interval; HPD, high population density; SARS, severe acute respiratory syndrome; hCoV, human coronavirus; GTR + Γ + I, general time reversible gamma-shaped rate distribution across sites and an invariant site assumption. †Estimation of the year (BC) of the most recent common ancestor. ‡Estimation of the year of the most recent common ancestor of extant CoV. All years AD except as indicated. §CoV group 2b without novel Bt-CoV from this study (Figure 2).

Because it has been suggested that codon-based evolutionary models may be preferred for Bayesian phylogenetic inference from protein-coding datasets (*27*), analyses on the 817-bp dataset were repeated by using the SRD06 substitution model in BEAST. This analysis did not yield a different substitution rate, but resulted in older resulting MRCA dates (Table 2). A Bayes factor test conducted in TRACER yielded a strong estimate of superiority of the codon-based model over the GTR + Γ4 + I model (log_10_ Bayes factor 139 [20 is highly significant]). To further optimize the prediction of MRCA dates, the constant population size assumption used in all analyses was exchanged against expansion growth or exponential growth assumptions. Both assumptions were predicted to fit the data better than the constant size model (Bayes factors 13.5 and 13.9). There was no difference between the expansion and exponential models (Bayes factor 0.34 in favor of expansion). The MRCA date of hCoV-229E and the GhanaBt-CoVGrp1 was 1686 (expansion) or 1800 (exponential growth). Table 2 summarizes the results. Figure 3 shows a dated phylogeny of coronaviruses with MRCAs according to the 2 last mentioned analyses.

### Recombination

To determine whether CoV recombination might play a role in the studied virus population, the structural nucleocapsid gene was amplified using 8 nested RT-PCR primer sets that had been designed on alignments of all available CoV group 1 nucleocapsid sequences. Using a similar approach, we also tested the same samples for CoV group 2 nucleocapsid sequences. Only group 1 RT-PCRs yielded fragments. These fragments could be combined into contiguous 1,030-nt sequences for Bt-CoV GhanaKwam 19 and 1,176 nt for Bt-CoV GhanaBoo 344. As shown in Figure 3, panel B, the resulting phylogenetic placement was exactly matching that of the *RdRp* fragments, giving no evidence of recombination between the *RdRp* region located in the middle of the genome and the nucleocapsid gene located at the extreme downstream end. Sequencing of the nucleocapsid gene of the GhanaBt-CoVGrp2 was not successful when we used 15 nested RT-PCRs designed on alignments of all available CoV 2b nucleocapsid sequences. Amplification with above mentioned nested RT-PCRs for CoV group 1 was also unsuccessful.

## Discussion

In the aftermath of the SARS epidemic, bats have been identified as carriers of CoV in China (*3**–**7*). Furthermore, in addition to our earlier finding of antibodies against CoVs in various African bats (*10*), we have confirmed the presence of CoV in bats of Ghana. Together with recent data from Germany, North America, Trinidad, and Kenya (*11**,**12**,**28*), these findings suggest that the association of CoV with bats is a worldwide phenomenon. The prevalence of CoV in insect-eating bats (9.76%) matched our previous findings in Germany. However, in that study we sampled during the breeding season and showed that CoVs are most likely amplified in maternity roosts (*11*). The composition of the catch in this study (no lactating females, no young bats) suggests sampling outside the breeding season and may not be directly comparable. Future studies relating to risks of exposure should address whether virus prevalence may change over time.

The risk of exposure was also addressed by investigations of virus concentration. Several groups have shown that CoVs are almost exclusively detected in bat feces and not, as hypothesized earlier, in saliva (*3**,**4**,**28**,**29*). Surprisingly, little virus was found in all fecal samples tested in our study. We estimated the RNA concentration per full sample (100 mg feces = 2–4 fecal pellets) to be only up to 4.5 × 10^4^ RNA copies. Human pathogenic viruses transmitted by the fecal-oral route generate much higher virus concentrations in stool, up to ≈10^12^ RNA copies/mg, e.g., for different picornaviruses (*30**–**32*). Based on these data it would be difficult to postulate that humans can acquire CoV from bat feces. However, studies in other locations and at different times are needed to address virus concentration in bat droppings in more detail. Because virus in this study was only observed in insectivorous bats and not in frugivorous bats, future studies should investigate whether insects might constitute a source of CoV infection for bats.

To achieve a direct prediction of the potential of bat CoVs to infect human cells, it would be highly relevant to conduct virus isolation studies on bat feces. However, in our study we sampled no more than 100 mg of feces per bat. All samples had to be collected in RNAlater solution (0.5 mL) (Applied Biosystems) for reasons of storage and transportation. Although it has been suggested that RNAlater solution may preserve virus infectivity (*33**,**34*), our observations showed that the solution has to be diluted at least 1:20 in cell culture medium to avoid cytotoxicity (data not shown). Because of the low virus RNA concentrations observed, we did not attempt to isolate the virus. However, the absence of successful virus isolation from bat feces in previous studies (*3**–**6**,**8**,**11**,**12*) may not reflect the incapability of bat CoV to infect human cells. Recently, a synthetic bat CoV complemented with an appropriate spike protein has shown potential to infect human cells (*35*).

Reconstruction of phylogenetic and temporal relationships between bat CoV and other mammalian CoV is another way to obtain information on their zoonotic potential. Unfortunately, for CoV long sequence fragments must be analyzed before valid phylogenies can be inferred from the conserved nonstructural genome portion (*28**,**36*). Because of the low concentration of RNA in bat samples, generation of long sequences from novel bat CoV is tedious and technically demanding, which may be why some published phylogenies of bat CoV are based on short datasets, making it difficult to use these data for reference. For molecular clock dating, we have therefore relied on reference viruses mainly from other mammals that covered our 1,221-bp fragment in the conserved *RdRp* region. We assumed that the *RdRp* would be under less selective pressure than the structural genes and other nonstructural genes, and therefore could be used to infer nucleotide substitution rates over distantly related CoVs (*7**,**25**,**26**,**36**–**38*). We have confirmed all tree topologies using alternative methods of phylogenetic inference, including an MCMC algorithm implemented in MrBayes that eliminates artifacts contributed by fixation of MCMC chains in suboptimal prosterior probability maxima (*20*). Calibration was conducted on reliable isolation dates of prototype and novel bat CoV from the literature, as well as on the MRCA of the hCoV-OC43/Bovine CoV clade. For dating of only this specific CoV clade, a wide range of dated virus isolates has been available that covered as much as 34% (1965–2004) of the projected time of virus evolution from root to tip (1890–2004) (*25*). A probabilistic calibration prior was used, which is favorable for dating in combination with relaxed molecular clock assumptions (*39*). The determined mean substitution rates were in good concordance with earlier studies on non-bat–CoV that used maximum likelihood-based methods in addition to Bayesian inference (*25**,**26**,**38**,**40*).

Although the exponential growth prior on the virus population seemed equivalent with an expansion growth model by the Bayes factor test and produced highly compatible MRCAs, the exponential model produced a better match with the previously determined MRCA of the HCoV-NL63/HCoV-229E pair (*26*). Because Pyrc et al. generated these data by 3 alternative approaches (Bayesian, serial unweighted pair group method with arithmetic mean, maximum likelihood [*26*]), we used their MRCA to validate our results, and consequently prefer the MRCA dating from the exponential growth population model (as presented in Figure 3 in plain type). One earlier study on bat and non-bat CoV suggested a much faster evolutionary rate for CoV than other studies (*7*). As Vijaykrishna et al. pointed out, their results were associated with large confidence intervals caused by the lack of available data on Bt CoV at the time the study was conducted (*7*). The increase of available sequence data now enables a better account of CoV evolutionary history.

All CoVs in our study were found in members of the genus *Hipposideros* (family Hipposideridae). The genus *Rhinolophus* from the sister family Rhinolophidae was found to host SARS-like viruses in several studies in China. One of our *Hipposideros* CoVs was in a basal phylogenetic relationship with the SARS-like clade (group 2b); their most recent common ancestors date back to ≈400 bc. Tong et al. (*17*) have detected a sequence fragment of a bat CoV in Kenya that also belongs to the 2b clade but is associated with the genus *Chaerephon*, a free-tailed bat that is rather distantly related to the genus *Hipposideros*. Although these authors analyzed only a short sequence fragment, their 2b CoV seems to be related more closely to SARS CoV than the virus found in our study. In the many studies conducted in China, only closely related members of the 2b group were detected, with the most basal members dating back only to the 17th century, according to our analysis. The co-occurrence of basal and closely related viruses in Africa, as well as the existence of the same virus clade in bats other than those of the family Hipposideridae, may entail speculations about a possible origin of the SARS-like group of CoVs in Africa rather than in Asia.

Another result that should be integrated with earlier findings is the surprisingly recent date of the MRCA of the novel Grp1 Bt CoV and the human common cold virus hCoV-229E. Further to the proven recent host switching of SARS CoV, Vijgen et al. have suggested that hCoV-OC43 entered the human population ≈120 years ago, causing a pandemic (*25*). This virus was most likely acquired by humans from domestic cattle. Results of our study show that it is not unlikely that hCoV-229E, which today is circulating worldwide in humans, resulted from a host switching event not more than 208–322 years ago. However, as with molecular clock dating of viruses, associated confidence limits should not be overlooked.

Because *H. cf. ruber* bats are found only in sub-Saharan Africa and are not migratory (*23*), it would be relevant to know how tightly the associated CoV is restricted to its host. Despite the statistical limitations of our rather small sample size, the absence of CoV in bats of the closely related species *H. abae* that were tested in our study in two different caves speaks in favour of tight host restriction. Another supportive argument is the absence of CoV in *C. afra*, a bat species sampled in sufficient numbers at the Booyem cave. This cave was coinhabited by CoV-positive *H. cf. ruber* bats. If tight host restriction to nonmigratory *H. cf. ruber* bats existed, this would indicate an origin of hCoV-229E within the geographic range of its host, i.e., the rainforest belt and the wet forested savannahs of sub-Saharan Africa (*23*). Unfortunately, it will be difficult to reconstruct whether the projected host transition event might have been associated with human epidemic disease.

## References

[1] Cavanagh D. Nidovirales: a new order comprising *Coronaviridae* and *Arteriviridae.* Arch Virol. 1997;142:629–33. 9349308

[2] Drosten C, Gunther S, Preiser W, van der Werf S, Brodt HR, Becker S, Identification of a novel coronavirus in patients with severe acute respiratory syndrome. N Engl J Med. 2003;348:1967–76. 10.1056/NEJMoa030747 12690091

[3] Lau SK, Woo PC, Li KS, Huang Y, Tsoi HW, Wong BH, Severe acute respiratory syndrome coronavirus-like virus in Chinese horseshoe bats. Proc Natl Acad Sci U S A. 2005;102:14040–5. 10.1073/pnas.0506735102 16169905 PMC1236580

[4] Li W, Shi Z, Yu M, Ren W, Smith C, Epstein JH, Bats are natural reservoirs of SARS-like coronaviruses. Science. 2005;310:676–9. 10.1126/science.1118391 16195424

[5] Poon LL, Chu DK, Chan KH, Wong OK, Ellis TM, Leung YH, Identification of a novel coronavirus in bats. J Virol. 2005;79:2001–9. 10.1128/JVI.79.4.2001-2009.2005 15681402 PMC546586

[6] Tang XC, Zhang JX, Zhang SY, Wang P, Fan XH, Li LF, Prevalence and genetic diversity of coronaviruses in bats from China. J Virol. 2006;80:7481–90. 10.1128/JVI.00697-06 16840328 PMC1563713

[7] Vijaykrishna D, Smith GJ, Zhang JX, Peiris JS, Chen H, Guan Y. Evolutionary insights into the ecology of coronaviruses. J Virol. 2007;81:4012–20. 10.1128/JVI.02605-06 17267506 PMC1866124

[8] Woo PC, Lau SK, Li KS, Poon RW, Wong BH, Tsoi HW, Molecular diversity of coronaviruses in bats. Virology. 2006;351:180–7. 10.1016/j.virol.2006.02.041 16647731 PMC7111821

[9] Wong S, Lau S, Woo P, Yuen KY. Bats as a continuing source of emerging infections in humans. Rev Med Virol. 2007;17:67–91. 10.1002/rmv.520 17042030 PMC7169091

[10] Muller MA, Paweska JT, Leman PA, Drosten C, Grywna K, Kemp A, Coronavirus antibodies in African bat species. Emerg Infect Dis. 2007;13:1367–70. 18252111 10.3201/eid1309.070342 PMC2857293

[11] Gloza-Rausch F, Ipsen A, Seebens A, Gottsche M, Panning M, Felix Drexler J, Detection and prevalence patterns of group I coronaviruses in bats, northern Germany. Emerg Infect Dis. 2008;14:626–31. 10.3201/eid1404.071439 18400147 PMC2570906

[12] Dominguez SR, O’Shea TJ, Oko LM, Holmes KV. Detection of group 1 coronaviruses in bats in North America. Emerg Infect Dis. 2007;13:1295–300. 18252098 10.3201/eid1309.070491 PMC2857301

[13] Proches S. The world’s biogeographical regions: cluster analyses based on bat distributions. J Biogeogr. 2005;32:607–14. 10.1111/j.1365-2699.2004.01186.x

[14] Mayer F, Dietz C, Kiefer A. Molecular species identification boosts bat diversity. Front Zool. 2007;4:4. 10.1186/1742-9994-4-4 17295921 PMC1802075

[15] Calisher CH, Childs JE, Field HE, Holmes KV, Schountz T. Bats: important reservoir hosts of emerging viruses. Clin Microbiol Rev. 2006;19:531–45. 10.1128/CMR.00017-06 16847084 PMC1539106

[16] Swensson J. Bushmeat trade in Techiman, Ghana, West Africa. Uppsala (Sweden): Uppsala University; 2005.

[17] Tong S, Conrardy C, Ruone S, Kuzmin IV, Guo X, Tao Y, Detection of novel SARS-like and other coronaviruses in bats from Kenya. Emerg Infect Dis. 2009;15:482–5. 10.3201/eid1503.081013 19239771 PMC2681120

[18] de Souza Luna LK, Heiser V, Regamey N, Panning M, Drexler JF, Mulangu S, Generic detection of coronaviruses and differentiation at the prototype strain level by reverse transcription-PCR and nonfluorescent low-density microarray. J Clin Microbiol. 2007;45:1049–52. 10.1128/JCM.02426-06 17229859 PMC1829107

[19] Tamura K, Dudley J, Nei M, Kumar S. MEGA4: Molecular Evolutionary Genetics Analysis (MEGA) software version 4.0. Mol Biol Evol. 2007;24:1596–9. 10.1093/molbev/msm092 17488738

[20] Ronquist F, Huelsenbeck JP. MrBayes 3: Bayesian phylogenetic inference under mixed models. Bioinformatics. 2003;19:1572–4. 10.1093/bioinformatics/btg180 12912839

[21] Gelman A, Rubin DB. Markov chain Monte Carlo methods in biostatistics. Stat Methods Med Res. 1996;5:339–55. 10.1177/096228029600500402 9004377

[22] Drummond AJ, Rambaut A. BEAST: Bayesian evolutionary analysis by sampling trees. BMC Evol Biol. 2007;7:214. 10.1186/1471-2148-7-214 17996036 PMC2247476

[23] Vallo P, Guillén-Servent A, Benda P, Pires DB, Koubek P. Variation of mitochondrial DNA reveals high cryptic diversity in *Hipposideros caffer* complex. Acta Chiropt. 2008;10:193–206. 10.3161/150811008X414782

[24] Woo PC, Wang M, Lau SK, Xu H, Poon RW, Guo R, Comparative analysis of twelve genomes of three novel group 2c and group 2d coronaviruses reveals unique group and subgroup features. J Virol. 2007;81:1574–85. 10.1128/JVI.02182-06 17121802 PMC1797546

[25] Vijgen L, Keyaerts E, Moes E, Thoelen I, Wollants E, Lemey P, Complete genomic sequence of human coronavirus OC43: molecular clock analysis suggests a relatively recent zoonotic coronavirus transmission event. J Virol. 2005;79:1595–604. 10.1128/JVI.79.3.1595-1604.2005 15650185 PMC544107

[26] Pyrc K, Dijkman R, Deng L, Jebbink MF, Ross HA, Berkhout B, Mosaic structure of human coronavirus NL63, one thousand years of evolution. J Mol Biol. 2006;364:964–73. 10.1016/j.jmb.2006.09.074 17054987 PMC7094706

[27] Shapiro B, Rambaut A, Drummond AJ. Choosing appropriate substitution models for the phylogenetic analysis of protein-coding sequences. Mol Biol Evol. 2006;23:7–9. 10.1093/molbev/msj021 16177232

[28] Carrington CV, Foster JE, Zhu HC, Zhang JX, Smith GJ, Thompson N, Detection and phylogenetic analysis of group 1 coronaviruses in South American bats. Emerg Infect Dis. 2008;14:1890–3. 10.3201/eid1412.080642 19046513 PMC2634629

[29] Dobson AP. Virology. What links bats to emerging infectious diseases? Science. 2005;310:628–9. 10.1126/science.1120872 16254175

[30] Baumgarte S, de Souza Luna LK, Grywna K, Panning M, Drexler JF, Karsten C, Prevalence, types, and RNA concentrations of human parechoviruses, including a sixth parechovirus type, in stool samples from patients with acute enteritis. J Clin Microbiol. 2008;46:242–8. 10.1128/JCM.01468-07 18057123 PMC2224249

[31] Drexler JF, Luna LK, Stocker A, Almeida PS, Ribeiro TC, Petersen N, Circulation of 3 lineages of a novel Saffold cardiovirus in humans. Emerg Infect Dis. 2008;14:1398–405. 10.3201/eid1409.080570 18760006 PMC2603095

[32] Takanashi S, Hashira S, Matsunaga T, Yoshida A, Shiota T, Tung PG, Detection, genetic characterization, and quantification of norovirus RNA from sera of children with gastroenteritis. J Clin Virol. 2009;44:161–3. 10.1016/j.jcv.2008.11.011 19131272

[33] Forster JL, Harkin VB, Graham DA, McCullough SJ. The effect of sample type, temperature, and RNAlater on the stability of avian influenza virus RNA. J Virol Methods. 2008;149:190–4. 18294703 10.1016/j.jviromet.2007.12.020

[34] Uhlenhaut C, Kracht M. Viral infectivity is maintained by an RNA protection buffer. J Virol Methods. 2005;128:189–91. 10.1016/j.jviromet.2005.05.002 15936833

[35] Becker MM, Graham RL, Donaldson EF, Rockx B, Sims AC, Sheahan T, Synthetic recombinant bat SARS-like coronavirus is infectious in cultured cells and in mice. Proc Natl Acad Sci U S A. 2008;105:19944–9. 10.1073/pnas.0808116105 19036930 PMC2588415

[36] Snijder EJ, Bredenbeek PJ, Dobbe JC, Thiel V, Ziebuhr J, Poon LL, Unique and conserved features of genome and proteome of SARS-coronavirus, an early split-off from the coronavirus group 2 lineage. J Mol Biol. 2003;331:991–1004. 10.1016/S0022-2836(03)00865-9 12927536 PMC7159028

[37] Bredenbeek PJ, Snijder EJ, Noten FH, den Boon JA, Schaaper WM, Horzinek MC, The polymerase gene of corona- and toroviruses: evidence for an evolutionary relationship. Adv Exp Med Biol. 1990;276:307–16. 1966417 10.1007/978-1-4684-5823-7_42

[38] Salemi M, Fitch WM, Ciccozzi M, Ruiz-Alvarez MJ, Rezza G, Lewis MJ. Severe acute respiratory syndrome coronavirus sequence characteristics and evolutionary rate estimate from maximum likelihood analysis. J Virol. 2004;78:1602–3. 10.1128/JVI.78.3.1602-1603.2004 14722315 PMC321409

[39] Drummond AJ, Ho SY, Phillips MJ, Rambaut A. Relaxed phylogenetics and dating with confidence. PLoS Biol. 2006;4:e88. 10.1371/journal.pbio.0040088 16683862 PMC1395354

[40] Sanchez CM, Gebauer F, Sune C, Mendez A, Dopazo J, Enjuanes L. Genetic evolution and tropism of transmissible gastroenteritis coronaviruses. Virology. 1992;190:92–105. 10.1016/0042-6822(92)91195-Z 1326823 PMC7131265

